# Unveiling the Uncommon: Bilateral Absence of Musculocutaneous Nerve in Cadaveric Examination

**DOI:** 10.7759/cureus.91133

**Published:** 2025-08-27

**Authors:** Rachel P Seng, Tsolaye Ugbeye, Uver Rodriguez-Argueta, Nicole Vlahos, Landon J Zachary, Nora Hassan, Joseph Bogaudo, Jason A Brown II, Selina Hanna, Kavita A Gopal, Parker Velek, Raymond Colello

**Affiliations:** 1 Anatomy, American University of the Caribbean, Cupecoy, SXM; 2 Neurology, American University of the Caribbean, Sint Maarten, MAF

**Keywords:** anatomical variability, brachial plexus, brachial plexus anatomic variation, musculocutaneous nerves, musculocutaneous nerve variations, upper limb surgery

## Abstract

The brachial plexus is a network of nerves crucial to the innervation of the upper limb. The anatomy of the brachial plexus has been thought to be consistent across persons and is studied as such. Instances of deviations of this plexus pose significant challenges in both clinical diagnosis and surgical interventions. The musculocutaneous nerve, originating from the lateral cord of the brachial plexus, allows for movement of key muscles of the arm and cutaneous sensation to the forearm. Our present case reveals a unique bilateral absence of the musculocutaneous nerve. We aim to elucidate the anatomical, clinical, and surgical implications of a rare bilateral absence and compare our findings to similar reports.

## Introduction

A lab at the American University of the Caribbean discovered a bilateral absence of the musculocutaneous nerve (MCN) in a cadaver. The MCN is one of the terminal branches of the lateral cord of the brachial plexus. Originating from spinal nerves C5, C6, and C7, its path runs through the axilla and inserts into the coracobrachialis. The MCN innervates the brachialis, biceps brachii, and the coracobrachialis muscles. Passing laterally to the tendon of the biceps brachii, the MCN gives rise to the lateral antebrachial cutaneous nerve, providing sensation over the lateral forearm and motor innervation of the anterior compartment of the arm [1]. Any anatomic abnormality has surgical implications, which makes continued research crucial to determine what structures are compensating for motor innervations, cutaneous sensation, and to trace the compensating nerve's new pathway. This article was previously presented as a poster presentation at the Spring 2024 American University of the Caribbean semesterly Research Symposium on March 01, 2024.

## Case presentation

Identification of the anatomical anomalies occurred during class dissections, prompting the decision to proceed with further investigations. Subsequent steps involved conducting additional dissections to enhance the visualization of the nerve pathways. Following the clearance of anatomical structures, researchers meticulously traced the nerves from their origins through the branching points to determine their innervation targets, documenting the associated muscles and skin areas. Various tools, including probes, scalpels, and scissors, were used as needed to facilitate dissection.

Documentation involved capturing photographs of the brachial plexus and its innervations bilaterally to highlight both abnormal and normal features of the cadaver. A few images were selected and labeled by the researchers.

The MCN is one of the terminal branches of the brachial plexus that supplies motor and sensory innervation to the anterior compartment of the arm and lateral forearm. Originating from the C5 to C7 spinal nerve roots, it is formed by the union of the superior and middle trunk, where it divides into the lateral cord before branching into the MCN [1]. After its separation from the lateral cord, the MCN pierces the coracobrachialis, where it supplies motor innervation. By innervating the coracobrachialis, the nerve plays a role in flexion and vertical adduction of the glenohumeral joint. It further supplies motor innervation to both heads of the biceps brachii and the brachialis. The biceps brachii allows for flexion and supination of the elbow, while the brachialis only performs flexion [2]. The MCN is also documented as giving rise to the lateral cutaneous nerve of the forearm, where it provides sensory innervation to the lateral compartment [3,4].

There have been extensive reports of variations of the MCN such as deviations in anatomical location, complete absence, and partial absence. One of the most common documented variations occurs where the nerve penetrates the coracobrachialis. Often these variations are silent and only discovered postmortem. As reported, the variations of the nerves from the lateral cord may be due to altered signaling between the axons of spinal nerves, as they grow toward the mesenchyme around the fifth week of embryological development. The mechanism by which this occurs is not well understood [5]. 

Anatomical variations are important to consider in all care. Within the brachial plexus, five spinal nerves join and separate into various nerves. Variations occur during the branching process of these nerves. In this case report, the absence of the MCN is observed bilaterally, and an additional anatomical variation is found on the right arm involving a lack of the entire posterior cord. A review of the same anatomy found that this anatomical variation leads to subsequent deviation in innervation of the biceps brachii, brachialis, and (if present) the accessory head of the biceps brachii [6]. Upon examination, it was determined that innervation instead stems from the median nerve. This helps to solidify that even in the absence of typical innervation, the median nerve can compensate. The median nerve does not contribute to the arm in the typical anatomical presentation. Instead, it is responsible for the sensation of the forearm extending from the elbow to the wrist through an anterior and posterior branch on the radial border [3]. Here, it is responsible for the forearm pronators and flexors, with the exception of the flexor carpi ulnaris and the ulnar aspect of the flexor digitorum profundus, the skin on the palm and fingers, along with some muscles of the hand [6]. Injury to the median nerve in patients with typical anatomy presents with sensory and motor function deficits to the hand and wrist. The review noted that in patients with MSN absence, injury occurring in early segments of the median nerve likely presents with clinical manifestations in the upper portion of the limb. In addition to the typical symptoms, there is a record of weak shoulder flexion, elbow flexion, and supination, along with lateral forearm cutaneous sensory deficit [7]. In the study conducted over the span of eight years, 80 limbs were surveyed at a medical college. Two limbs from separate cadavers were documented with MCN abnormalities: one with a communication between the MCN and median nerve, and the other with an MCN that does not pierce the coracobrachialis [3].

Atypical presentations and innervations can make for complicated cases with surgery intervention. In the fixation of a humerus, the identification of the median nerve and MCN intraoperatively can cause confusion if the patient presents with an absent MCN [6]. Further, communications of the MCN and median nerve can also exist with anatomical diversity [8]. A study summarizes the categorization of the MCN and the median nerve communications into five different categories (Table 1) [8].

**Table 1:** Jamuna and Amudha categorization of documented variations of the musculocutaneous nerve

Variation	Description of anatomy
Type I	There is no communication between the median nerve and the musculocutaneous nerve.
Type II	The fibers of the lateral root of the median nerve pass through the musculocutaneous nerve and join the median nerve in the middle of the arm.
Type III	The lateral root fibers of the median nerve pass along the musculocutaneous nerve and, after some distance, leave it to form the lateral root of the median nerve.
Type IV	The musculocutaneous nerve fibers join the lateral root of the median nerve, and after some distance, the musculocutaneous nerve arises from the median nerve.
Type V	The musculocutaneous nerve is absent, and all fibers are directed through the lateral root. Muscles are innervated directly from the median nerve.

Abnormalities of the cords of the brachial plexus have also been noted in the literature. In a survey of 172 cadavers, 3.5% were found to lack the posterior cord [9]. In another review of 173 cadavers with cord abnormalities, an absence of a posterior cord was observed in 36 [10]. Proper documentation of these anomalies provides a clearer understanding of how common variations might actually be.

Anatomical variations are theorized to be attributed to many factors. An article states that the revolving nerve growth is both an attractant and a repelling force. It is summarized that embryological signaling in the fifth week impacts the development and formation of the brachial plexus. Furthermore, insufficient signaling does not allow for the propagation of spinal nerves to reach the mesenchyme of the limb, leading to abnormal variations and atypical anatomical presentation [11].

During a standard cadaveric dissection of a male, a bilateral absence of the MCN was identified. The exact age of the subject before passing is unknown, but it appears to be advanced. While a bilateral absence is already noted as uncommon, each arm portrayed different configurations, raising further questions. 

**Figure 1:**
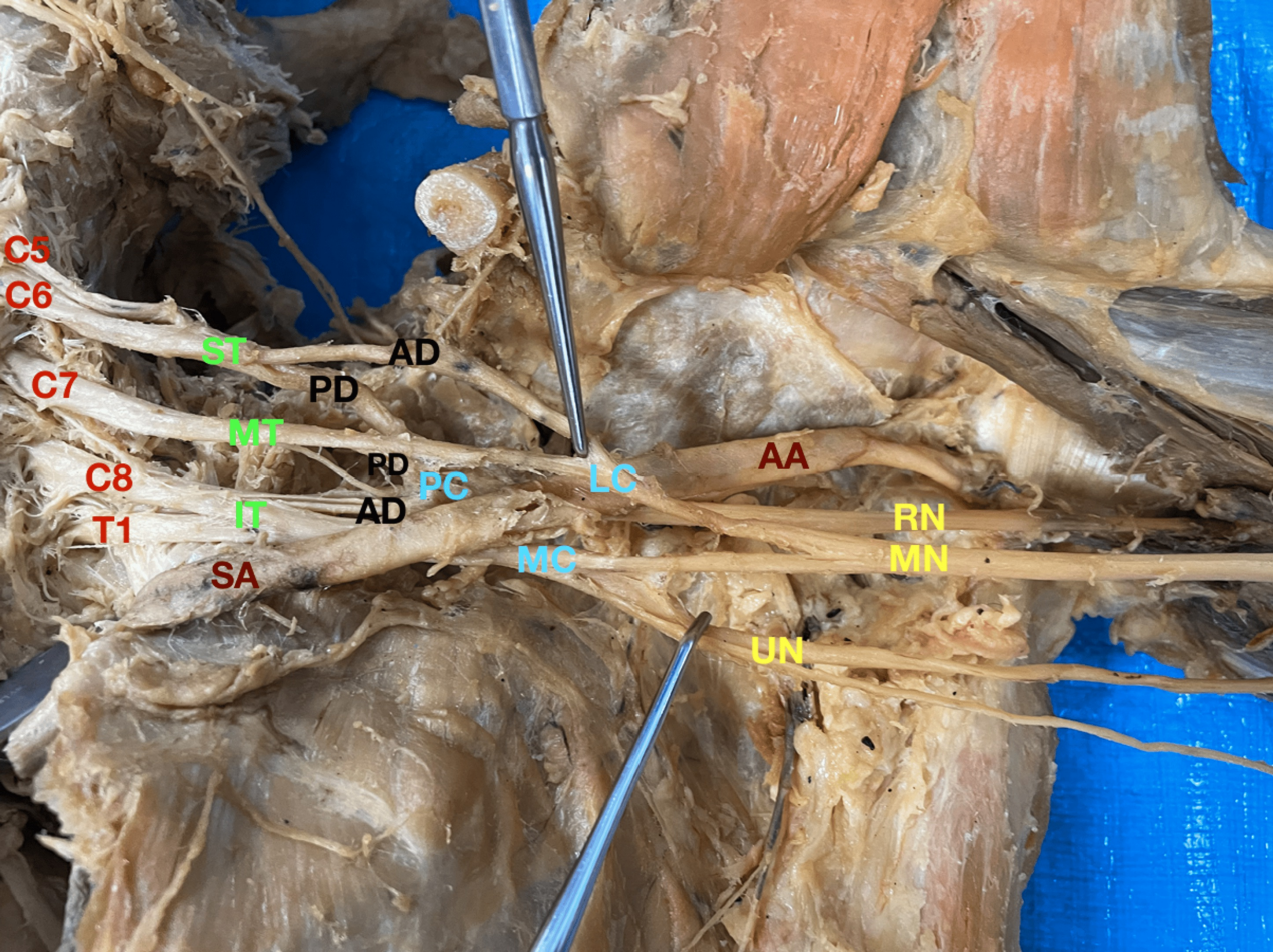
Left arm abnormalities Red: Roots; Dark red: Vessels; Green: Trunks; Black: Divisions; Blue: Cords; Yellow: Branches. AA: Axillary artery; AD: Anterior division; AN: Axillary nerve; C5: Cervical spinal nerve root 5; C6: Cervical spinal nerve root 6; C7: Cervical spinal nerve root 7; C8: Cervical spinal nerve root 8; IT: Inferior trunk; LC: Lateral cord; MC: Medial cord; MN: Median nerve; MT: Middle trunk; PC: Posterior cord; PD: Posterior division; RN: Radial nerve; SA: Subclavian artery; SSN: Subscapular nerve; T1: Thoracic spinal nerve root 1; UN: Ulnar nerve.

The left arm displayed the simplest anomaly of the two, as it only contained an absence of the MCN (Figure 1). All other aspects of the brachial plexus remained intact in normal anatomical fashion. The median nerve was found to act as a surrogate nerve in the presence of an absent MCN. The lateral cutaneous nerve, which normally branches off the MCN, was instead fed by the median nerve.

**Figure 2:**
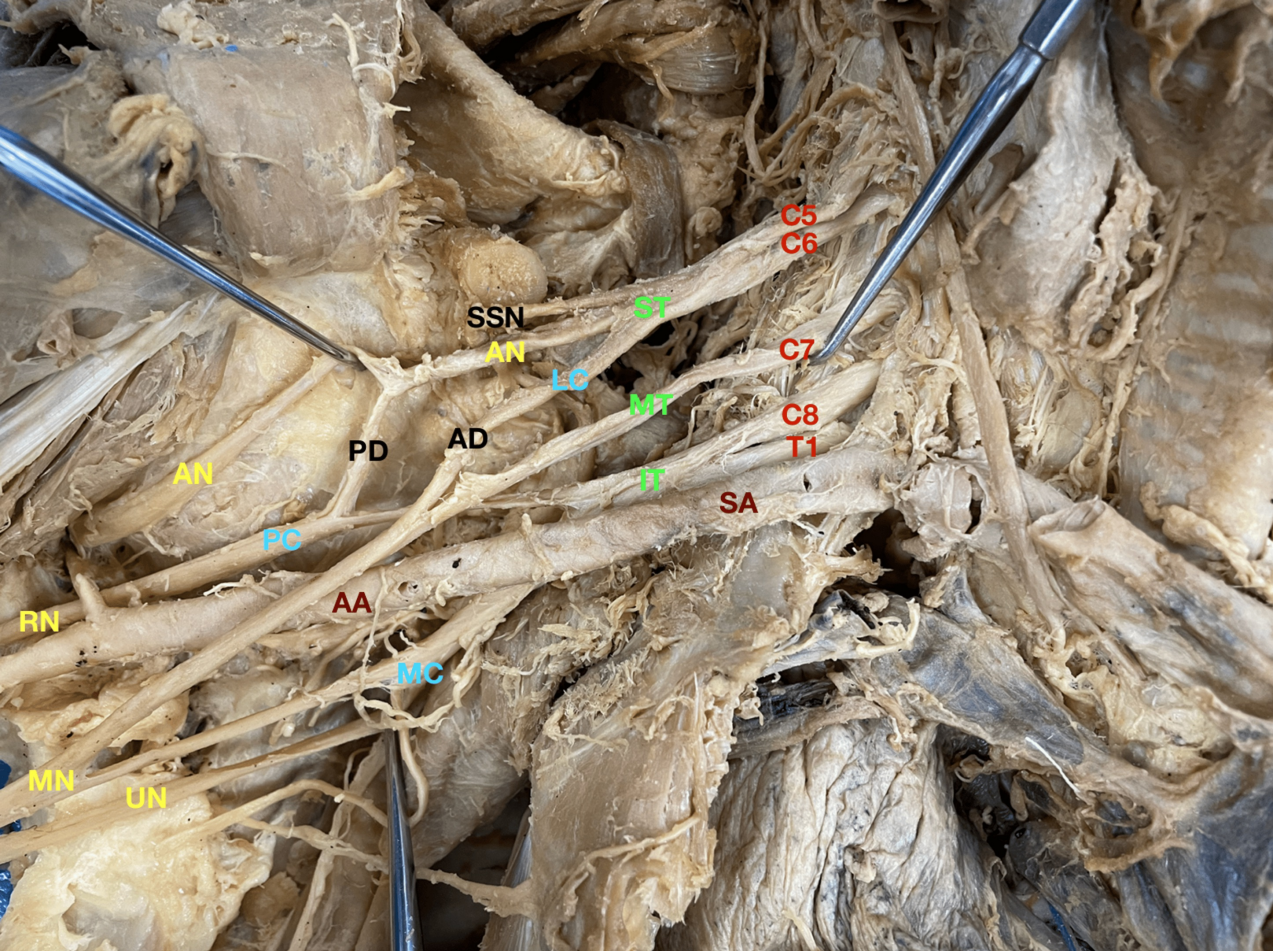
Right arm abnormalities Red: Roots; Dark red: Vessels; Green: Trunks; Black: Divisions; Blue: Cords; Yellow: Branches. AA: Axillary artery; AD: Anterior division; AN: Axillary nerve; C5: Cervical spinal nerve root 5; C6: Cervical spinal nerve root 6; C7: Cervical spinal nerve root 7; C8: Cervical spinal nerve root 8; IT: Inferior trunk; LC: Lateral cord; MC: Medial cord; MN: Median nerve; MT: Middle trunk; PC: Posterior cord; PD: Posterior division; RN: Radial nerve; SA: Subclavian artery; SSN: Subscapular nerve; T1: Thoracic spinal nerve root 1; UN: Ulnar nerve.

The right arm exhibited a much more complex arrangement. The brachial plexus on this side contained other abnormalities in addition to the absence of the MCN (Figure 2). Upon inspection, there appeared to be a complete lack of the posterior cord. In turn, the axillary nerve formed its branch solely off the superior cord (C5 and C6) instead of standard contributions from all roots of the brachial plexus (C5-T1). Abnormalities of the cords have also been noted in the literature. In a study reviewing 172 cadavers, 3.5% were found to lack a posterior cord [9].

## Discussion

As mentioned before, while most muscles of the upper extremity are innervated by multiple nerves, the coracobrachialis is solely innervated by the MCN. In both arms, it was observed that the biceps brachii, brachialis, and coracobrachialis were innervated by branches of the median nerve similar to previous cases [1-17]. While it is difficult to determine based on a lack of information regarding subjects' medical history and lifestyle, the muscles were noted as being well developed. Thus indicating evidence against muscle weakness and motor deficits. The branches of the median nerve that innervated the biceps brachii were found more distal than their typical proximal branches that provide innervation via the MCN. The median nerve also contributed to the cutaneous sensory innervation of the lateral forearm bilaterally, replacing the typical innervation supplied by the MCN, as described in prior documentation of anomalies [3]. With innervation being supplemented, it can be assumed that no sensory deficits of the lateral forearm were present premortem.

While documented in the literature, the absence of MCN is poorly understood and rarely observed in clinical practice [3,7,8,11,12]. Out of the historical cases reviewed, only three studies specifically mention a bilateral case of the MCN [6,12,16]. The first case from 2020 documents a singular bilateral absence in a 66-year-old female cadaver [6]. The second study, which has completed a meta-analysis of MCN absence occurrences, identifies five cadavers (two females, two males, and one of unknown sex) [12]. Finally, the third case briefly mentions a documentation of one case in 1998 with no further description [16]. These studies further exemplify the novelty of bilateral abnormalities as a subset of MCN variations. Other studies exhibiting MCN variations fail to specify whether the occurrences were unilateral or bilateral, leaving it to an assumption that the presentations were unilateral.

When the MCN is absent, the musculature of the arm requires innervation from a surrogate nerve. In the present study, the median nerve acted as a surrogate. Clinically, an injury of the median nerve will produce muscle weakness or paralysis of the flexor muscles of the arm. In the case of humerus fracture fixation, identification of the median nerve and the MCN is important, especially in its absence. This can lead to surgical confusion during fracture fixation [6]. In regard to nerve transfer surgeries, i.e., spinal accessory nerve, intercostal nerve, medial pectoral nerve, and ulnar nerve, surgeons should have an understanding of the anatomical possibilities before proceeding with surgical intervention. The decision to perform nerve transfers relies on intact and active branches that supply elbow flexors and can be avoided. An example of this is observed in a case report of a 42-year-old man who sustained a fall from a bike and developed left-sided brachial plexus injuries. The patient presented to the emergency department 11 months after the injury with no previous surgical management. Clinical examination included shoulder abduction, external rotation, and elbow flexion. Elbow extension exhibited weakness. Examination of the hand showed no pathology. Electrodiagnostic studies confirmed preganglionic nerve injury involving the C5 and C6 nerve roots. Patient underwent nerve transfer of the spinal accessory to the suprascapular nerve. However, further proximal dissection could not locate the MCN. Only the median nerve supplying the biceps muscle was identified. Nerve stimulation found proper bicep contraction, and nerve transfer was deferred [13]. 

Embryologically, any disturbances during the formation of limb muscles and peripheral nerves in early life, such as circulatory factors at the time of fusion of the brachial plexus cord, can be attributed to communication between the MCN and the median nerve [14-17]. This process occurs so early during embryological development, and the inability to test for these abnormalities makes medical intervention difficult. Ultimately, intervention would be drastically invasive, and since abnormalities such as these have so far been shown to be benign, there is no reason to do so [3]. Due to anomalies such as those historically documented in postmortem reports, limitations exist that require further investigation. Without live documented individuals, there is uncertainty whether these structural variations cause any physical deviations compared to those with the standard anatomy. These possibilities may include, but are not limited to, muscle weakness, diminished sensation, and referred pain resulting from compensatory innervation.

## Conclusions

An absent MCN can have many implications, and it is hypothesized that some of the most common symptoms resulting from an absent MCN could manifest as muscle weakness, paralysis, hypoesthesia of the lateral part of the forearm, functional impairment, altered reflexes, compensatory mechanisms from surrogate nerves, and pain or discomfort. Due to the suspected embryological origin, patients who contain these abnormalities are unable to differentiate between their motor and sensory function in comparison to those whose anatomy is altered surgically after injury. Almost every documented case of absent MCN has been found in postmortem examinations. This makes indicating the full effect of brachial plexus deviations difficult without the ability to conduct motor and sensory function tests.
